# Codon Usage of Hepatitis E Viruses: A Comprehensive Analysis

**DOI:** 10.3389/fmicb.2022.938651

**Published:** 2022-06-21

**Authors:** Bingzhe Li, Han Wu, Ziping Miao, Linjie Hu, Lu Zhou, Yihan Lu

**Affiliations:** ^1^Department of Epidemiology, Ministry of Education Key Laboratory of Public Health Safety, School of Public Health, Fudan University, Shanghai, China; ^2^Institute of Communicable Diseases Prevention and Control, Zhejiang Provincial Center for Disease Control and Prevention, Hangzhou, China

**Keywords:** hepatitis E virus, codon usage, relatively synonymous codon usage, effective codon number, zoonotic pathogen

## Abstract

Hepatitis E virus (HEV) is an emerging zoonotic pathogen with multiple species and genotypes, which may be classified into human, animal, and zoonotic HEV. Codon usage bias of HEV remained unclear. This study aims to characterize the codon usage of HEV and elucidate the main drivers influencing the codon usage bias. A total of seven HEV genotypes, HEV-1 (human HEV), HEV-3 and HEV-4 (zoonotic HEV), HEV-8, HEV-B, HEV-C1, and HEV-C2 (emerging animal HEV), were included in the study. Complete coding sequences, ORF1, ORF2, and ORF3, were accordingly obtained in the GenBank. Except for HEV-8, the other six genotypes tended to use codons ending in G/C. Based on the analysis of relatively synonymous codon usage (RSCU) and principal component analysis (PCA), codon usage bias was determined for HEV genotypes. Codon usage bias differed widely across human, zoonotic, and animal HEV genotypes; furthermore, it varied within certain genotypes such as HEV-4, HEV-8, and HEV-C1. In addition, dinucleotide abundance revealed that HEV was affected by translation selection to form a unique dinucleotide usage pattern. Moreover, parity rule 2 analysis (PR2), effective codon number (ENC)-plot, and neutrality analysis were jointly performed. Natural selection played a leading role in forming HEV codon usage bias, which was predominant in HEV-1, HEV-3, HEV-B and HEV-C1, while affected HEV-4, HEV-8, and HEV-C2 in combination with mutation pressure. Our findings may provide insights into HEV evolution and codon usage bias.

## Introduction

Hepatitis E virus (HEV) is a non-enveloped and quasi-enveloped positive-sense single-stranded RNA virus, approximately 27–30 nm in diameter (Yin et al., 2016). The HEV genome consists of three discontinuous open reading frames (ORF), of which ORF1 encodes non-structural proteins, ORF2 encodes viral capsid proteins, and ORF3 encodes a protein involved in the release of viral particles from infected cells (Ahmad et al., 2011). In addition, ORF4 has been identified in HEV *Orthohepevirus* A genotype 1 (HEV-1) and *Orthohepevirus* C, which is embedded entirely within ORF1. ORF4 may enhance the replication of HEV-1 in cell culture (Nair et al., 2016; Shafat et al., 2021). Genus *Orthohepevirus* in the family *Hepeviridae* includes all the HEV variants of mammals and birds so far discovered. HEV has been genetically classified into four species, *Orthohepevirus* A to D. In *Orthohepevirus* A (HEV-A), a total of eight genotypes have been identified, of which HEV-1 and HEV-2 are known to infect only humans (Khuroo and Khuroo, 2016). HEV-3 and HEV-4 are zoonotic pathogens with a wide range of animal hosts, among which swine is the most common host (Kenney, 2019). HEV-5 and HEV-6 are animal HEV solely isolated from wild boars in Japan (Takahashi et al., 2010). HEV-7 and HEV-8 are potential pathogens for infecting humans whose natural reservoirs are dromedaries (*Camelus dromedaries*) and bactrianus (*Camelus bactrianus*), respectively (Sridhar et al., 2017). *Orthohepevirus* C (HEV-C) has been mainly isolated in rats and ferrets and classified into two genotypes, C1 and C2 (HEV-C1 and HEV-C2). In the past, HEV-C has been considered to be incapable of infecting humans; however, multiple human cases with HEV-C infection have been documented, especially in Hong Kong in recent years (Sridhar et al., 2021).

Increasing species and genotypes of HEV has raised a new concern how the HEV variants evolve. It warrants further study in addition to phylogenetic analysis. Codon is the link between protein and nucleic acid, which plays a vital role in the transmission of genetic information. Among the 20 amino acids, except tryptophan and methionine having single codons, the other amino acids all have more than one encoding codons, which are defined as synonymous codons. However, the frequency of synonymous codon usage is not equal in the process of protein synthesis. A species or a gene usually tends to use one or more specific synonymous codons, called codon usage bias (He et al., 2019). It has been documented that the codon usage of some RNA viruses, such as hepatitis A virus, rotavirus, and SARS-CoV-2, has a stronger bias (Belalov and Lukashev, 2013; Pintó et al., 2018; Kandeel et al., 2020), while that of H7N9 influenza A virus, rabies virus, and atypical swine fever virus shows lower bias (Zhang et al., 2018; Pan et al., 2020; Sun et al., 2020). Codon usage bias between virus and host may be correlated with virus survival, adaptation, evolution, and immune escape (Bera et al., 2017). Therefore, codon usage bias can provide a deep understanding of molecular evolution and regulation of viral gene expression and facilitate the development of more effective vaccines. So far, however, the codon usage bias of HEV species and genotypes remains unclear.

Moreover, codon usage bias may be affected by some factors. Previous studies demonstrated that natural selection (such as in porcine epidemic diarrhea virus and human Bocavirus; Hussain et al., 2019; Yu et al., 2021), mutation pressure (such as in hepatitis C virus and Chikungunya virus; Hu et al., 2011; Butt et al., 2014), or both (such as in Japanese encephalitis virus and Banna virus; Singh et al., 2016; Long et al., 2018) mainly affect the codon usage bias of certain viruses. In addition, dinucleotide abundance, tRNA abundance, gene function and length may have influence (Zhang et al., 2018), which may further shape the codon usage bias. Therefore, in this study, we compared the codon usage patterns among human HEV (HEV-1), zoonotic HEV (HEV-3, HEV-4), and emerging animal HEV (HEV-8, HEV-C1, and HEV-C2), characterized their bias and explored the possible influence of mutation pressure and natural selection.

## Materials and Methods

### Data Collection

A total of 98 HEV complete genome sequences were retrieved in the NCBI GenBank database and then classified into HEV-1, HEV-3, HEV-4, HEV-8, HEV-B, HEV-C1, and HEV-C2.^1^ Considering that the number of complete genome sequences was too limited to form clustering and fitting curves, HEV-2 (*n* = 3), HEV-5 (*n* = 2), HEV-6 (*n* = 2), and HEV-7 (*n* = 3) were excluded from this study. All the genome sequences were managed and aligned using MEGA X (Kumar et al., 2018), and the ORFs with true start and end were extracted. In order to determine the overall codon usage bias, the HEV genomes were arranged in the following sequence: ORF1-ORF3-ORF2, after removing the stop codons. Accession numbers of the HEV genome sequences are listed in the Supplementary Table 1.

### Nucleotide Composition

The nucleotide composition of HEV complete genomes, ORF1, ORF2, and ORF3 coding sequences were calculated. Then the nucleotides at the third synonymous codon position (%A3s, %C3s, %T3s, and %G3s) and G + C nucleotide frequencies at the first, second and third codon sites (GC1s, GC2s, and GC3s) were estimated. GC values were calculated by online calculator.^2^ The correlation coefficients between nucleotide compositions (A%, T%, G%, C%, and GC%) and other nucleotide properties (A3%, T3%, G3%, C3%, and GC3%) of HEV complete coding sequences were calculated (Chakraborty et al., 2019) by Pearson’s method using R 4.1.1.^3^ A *p* < 0.05 was considered as statistically significant.

### Relative Synonymous Codon Usage

Relative synonymous codon usage (RSCU) is the ratio of a codon’s observed frequency and expected frequency encoding a particular amino acid (Wu et al., 2020). The expected frequency of codons is defined as the average number of codons encoding the amino acid. RSCU was calculated as follows:


(1) $$RSCU=\frac{Xij}{\sum_{j}^{ni}Xij}ni$$

where x_ij_ is the frequency of the j_th_ codon encoding the i_th_ amino acid, and n_i_ is the number of synonymous codons encoding the i_th_ amino acid. RSCU = 1 represents no codon usage bias; RSCU > 1 represents high codon usage frequency; and RSCU < 1 represents low codon usage frequency. In addition, RSCU > 1.6 or <0.6 is regarded as overrepresented or underrepresented codons, respectively (Wong et al., 2010). RSCU values were calculated by online calculator.^4^

### Principal Component Analysis

Principal component analysis (PCA) is a multivariate statistical method that reduces data dimensions by conducting covariance analysis between factors. In this study, RSCU values of the HEV genomes from different genotypes were converted into a small number of unrelated variables (called principal components) to study the major patterns and variations in codon usage. After excluding AUG, UGG, and three stop codons, the RSCU values of 59 synonymous codons in each coding sequence were distributed into a 59-dimensional vector. A matrix containing the 59 RSCU values in each sequence was constructed by PCA and then transformed into two axes. PCA was performed using the “psych” package 2.1.9 (Revelle, 2021) of R 4.1.1.

### Relative Dinucleotide Abundance Analysis

Relative dinucleotide abundance analysis was used to evaluate dinucleotide usage bias, as previously described (Karlin and Burge, 1995). Dinucleotide frequency was calculated as follows:


(2) $$\begin{array}{c} Pxy=\frac{fxy}{fxfy} \end{array}$$

where *fx* and *fy* represent the frequency of nucleotide *x* and *y*, respectively, and *fxy* represents the frequency of dinucleotide *xy*. When *fxy* was <0.78 or >1.23, the dinucleotide was considered underrepresented or overrepresented, respectively (Butt et al., 2016). The analysis was performed by using R 4.1.1.

### Parity Rule 2 Analysis

Parity Rule 2 (PR2) was used to investigate the effects of mutation and selection on codon usage. In the PR2 plot, AT deviation [A3/(A3 + T3)] and GC deviation [G3/(G3 + C3)] were selected as ordinate and abscissa, respectively. The center of the plot is defined as the origin coordinate (0.5, 0.5), which means A = U and G = C, indicating no deviation between mutation pressure and natural selection. The distance between scatter points and the center represents the degree of PR2 deviation (Sueoka, 1995). The analysis was performed by using R 4.1.1.

### Effective Number of Codons Analysis

Effective number of codon (ENC) is the number of effective codons used in a gene, reflecting the degree of preference for codon usage by a particular gene regardless of gene length (Wright, 1990). The ENC value ranges from 20 through 61. Codon usage bias is negatively correlated with the ENC value. Generally, the ENC value less than or equal to 45 indicates a high codon usage bias (Comeron and Aguadé, 1998). The ENC value was calculated as follows (Fuglsang, 2006):


(3) $$\begin{array}{c} \mathrm{ENC}=2+\frac{9}{F_{2}}+\frac{1}{F_{3}}+\frac{5}{F_{4}}+\frac{3}{F_{6}} \end{array}$$

where the average value of *F_i_* (*i* = 2, 3, 4, 6) for the *i*-fold degenerate amino acids is represented by *F*. The following formula was used to calculate *Fi* values:


(4) $$\begin{array}{c} F_{i}=\frac{n\sum_{j=1}^{i}{\left(\frac{n_{j}}{n}\right)}^{2}-1}{n-1} \end{array}$$

where the total number of appearances of the codons for that amino acid is represented by *n* and the total number of appearances of the *j_th_* codon for that amino acid is represented by *n_j_*. Furthermore, one-way analysis of variance (ANOVA) was utilized to test ENC difference among groups. Correlation between ENC values and GC contents (GC%, GC1%, GC2%, GC3%, and GC12%) was estimated by Pearson’s correlation (Chakraborty et al., 2019). A *p* < 0.05 was considered as statistically significant. The analysis was performed by using R 4.1.1.

### ENC-Plot Analysis

The ENC-plot analysis is commonly used to determine whether codon usage of genes is affected by natural selection (Wright, 1990). It is illustrated with the ENC values as ordinate and the GC3 values as abscissa. The theoretical ENC values were calculated as follows:


(5) $$\begin{array}{c} ENCexpected=2+GC3+\frac{29}{GC3^{2}+{\left(1-GC3\right)}^{2}} \end{array}$$

The expected ENC value falls on the theoretical curve when the codon usage bias is only affected by mutation pressure. When the actual ENC value falls below the curve, it indicates that the codon usage bias is affected by other factors, such as natural selection, in addition to mutation pressure (Pan et al., 2020). The analysis was performed by using R 4.1.1.

### Neutrality Plot Analysis and Correlation Analysis

The neutrality plot shows the effects of mutation pressure and natural selection on codon usage bias. Synonymous mutations generally occur at the third position of amino acid codons, while those at the first and second positions usually change the amino acid. When there is no external pressure, mutations occur randomly at three codon positions. The GC content at the third synonymous codon position (GC3) were plotted against the average GC content at the first and second synonymous codon positions (GC12). A linear fitting was performed on the plot. If the slope is closer to 1, the codon usage is mainly determined by mutation pressure. If the slope is closer to zero, it indicates natural selection that plays a greater role in the codon usage bias (Sueoka, 1988). Correlation analysis was utilized to test the slope of GC12 to GC3. A *p* < 0.05 was considered as statistically significant. The analysis was performed by using R 4.1.1.

### Relative Codon Deoptimization Index

We performed relative codon deoptimization index (RCID) to compare the similarities in codon usage between HEV genomes and their hosts (Mueller et al., 2006). RCDI values provide an insight into the rate of viral gene translation in a host genome. When RCDI values are close to 1, it means higher similarity in codon usage between a pathogen and its host, indicating greater adaptation of the pathogen to the host (Khandia et al., 2019). We selected several representative HEV hosts for comparison. RCDI values were calculated using http://genomes.urv.es/CAIcal (Puigbò et al., 2010). One-way ANOVA and Tukey’s HSD test were used to compare RCID values between HEV coding sequences and hosts using R 4.1.1. A *p* < 0.05 was considered as statistically significant.

### Ethical Approval

This study was approved by the Institutional Review Board (IRB) of the Fudan University School of Public Health (IRB 00002408 and FWA 00002399) under IRB #2021-04-0892. The study involved the use of existing sequence data in the online GenBank database. All data included in this study were without identifiers of humans or animals. No additional data was collected independently for this study. There was no need of obtaining informed consent.

## Results

### Nucleotide Composition of HEV ORF1, ORF2, ORF3, and Complete Genome

The proportion of GC3 in HEV-1, HEV-3, HEV-4, HEV-B, HEV-C1, and HEV-C2 were greater than 50% in complete genomes, indicating a preference for using codons ending in G/C (Table 1). Of them, HEV-B showed the largest codon usage bias. However, HEV-8 demonstrated a slight codon usage bias in complete genomes with the proportion of GC3 close to 50% and a minor tendency to use codons ending in A/U. In addition, the proportion of GC1 was the highest, while that of GC2 was the lowest in six genotypes, except HEV-8. Nucleotide composition of synonymous codons at the third position showed that the frequencies of G3 and C3 were higher than A3 and U3 (Table 1).

**Table 1:** Nucleotide composition of complete genomes in seven HEV genotypes.

Genotype	GC1^*^	GC2	GC3	A3s	U3s	C3s	G3s
HEV-B	64.68 ± 0.46	48.97 ± 0.15	55.89 ± 0.83	17.62 ± 1.21	33.95 ± 1.15	31.44 ± 0.78	36.65 ± 0.65
HEV-C1	62.8 ± 1.53	50.80 ± 3.14	57.62 ± 6.03	17.73 ± 3.38	31.74 ± 5.89	33.74 ± 2.48	35.97 ± 5.64
HEV-C2	55.78 ± 2.07	55.13 ± 2.73	55.46 ± 0.88	22.87 ± 1.97	27.82 ± 3.55	29.86 ± 0.85	35.89 ± 0.84
HEV-1	64.49 ± 0.23	51.27 ± 0.16	60.23 ± 0.52	10.87 ± 0.39	34.46 ± 0.67	41.77 ± 0.69	29.70 ± 0.46
HEV-3	63.45 ± 0.42	50.77 ± 0.27	54.81 ± 1.09	14.98 ± 0.73	37.03 ± 1.08	35.20 ± 1.13	30.55 ± 0.79
HEV-4	61.78 ± 3.81	52.20 ± 3.30	54.21 ± 3.70	17.19 ± 4.00	35.21 ± 7.99	33.25 ± 1.35	30.99 ± 3.92
HEV-8	60.70 ± 4.03	50.31 ± 0.42	49.89 ± 5.61	16.63 ± 3.89	40.22 ± 10.91	31.18 ± 0.87	27.71 ± 5.34

* The values in the cells were represented as mean% ± SD%.

Analysis of ORF1 and ORF2 provided similar findings with the complete genomes. In contrast, the proportion of GC3 in HEV-8 ORF1 was greater than 50%, while that of ORF2 was less than 50%, which was inconsistent with other six HEV genotypes. Analysis of ORF3 showed higher GC3 values than that of ORF1 and ORF2, indicating higher codon usage bias (Supplementary Table 2).

Furthermore, correlation coefficients were calculated between nucleotide compositions (A%, T%, G%, C%, and GC%) and other nucleotide properties (A3%, T3%, G3%, C3%, and GC3%) of HEV complete coding sequences (Table 2). Significant correlation was found between them at *p* < 0.01 or *p* < 0.05, indicating mutation pressure played a role in shaping the codon usage bias (Chakraborty et al., 2019).

**Table 2:** Correlation analysis between nucleotide composition and that at the third codon position of HEV complete coding sequences.

Genotype	Correlation	A3%	T3%	G3%	C3%	GC3%
HEV-1	A%	0.93^**^	−0.45	−0.75^*^	0.4	−0.04
	T%	−0.69^*^	0.92^**^	0.61	−0.88^**^	−0.64^*^
	G%	−0.96^**^	0.56	0.91^**^	−0.56	−0.06
	C%	0.65^*^	−0.92^**^	−0.63^*^	0.91^**^	0.67^*^
	GC%	0.31	−0.87^**^	−0.32	0.86^**^	0.82^**^
HEV-3	A%	0.95^**^	0.05	−0.72^**^	−0.16	−0.55^*^
	T%	−0.1	0.97^**^	−0.08	−0.79^**^	−0.79^**^
	G%	−0.57^*^	−0.14	0.97^**^	−0.09	0.44
	C%	−0.13	−0.9^**^	−0.1	0.94^**^	0.85^**^
	GC%	−0.43	−0.84^**^	0.46^*^	0.75^**^	0.96^**^
HEV-4	A%	0.31	−0.31	0.19	−0.02	0.26
	T%	−0.06	0.05	0.09	−0.4	−0.05
	G%	−0.2	0.25	−0.15	−0.03	−0.23
	C%	−0.04	0.03	−0.14	0.42^*^	0
	GC%	−0.14	0.15	−0.21	0.39	−0.12
HEV-8	A%	0.26	−0.05	0.02	−0.86^**^	−0.07
	T%	0.09	0.11	−0.13	−0.85^**^	−0.23
	G%	−0.81^*^	0.67	−0.65	0.81^*^	−0.56
	C%	0.31	−0.5	0.53	0.67	0.61
	GC%	−0.17	−0.05	0.09	0.93^**^	0.19
HEV-B	A%	0.97^**^	−0.59^*^	−0.39	−0.22	−0.42
	T%	−0.70^**^	0.92^**^	0.25	−0.52	−0.3
	G%	−0.26	−0.11	0.80^**^	−0.01	0.45
	C%	−0.38	−0.19	−0.19	0.88^**^	0.66^**^
	GC%	−0.49	−0.23	0.23	0.83^**^	0.86^**^
HE-C1	A%	0.47	0.8^**^	−0.76^**^	−0.7^**^	−0.86^**^
	T%	0.03	0.98^**^	−0.56^*^	−0.97^**^	−0.82^**^
	G%	−0.2	−0.98^**^	0.68^**^	0.9^**^	0.88^**^
	C%	−0.16	−0.98^**^	0.64^*^	0.98^**^	0.88^**^
	GC%	−0.18	−1^**^	0.67^**^	0.96^**^	0.89^**^
HEV-C2	A%	−0.11	0.26	0.08	−0.32	−0.52
	T%	0.33	−0.18	0.55	−0.75^*^	−0.13
	G%	0.19	−0.05	0.48	−0.68^*^	−0.22
	C%	−0.2	0.04	−0.45	0.67^*^	0.26
	GC%	−0.19	0.03	−0.41	0.63	0.27

** *p* < 0.01;

* *p* < 0.05.

### RSCU Patterns Across the HEV Genotypes

A total of 13 codons were preferably used by the seven HEV genotypes (RSCU>1; GCU[Ala], GCC[Ala], UGC[Cys], GAG[Glu], GGC[Gly], AAG[Lys], CUG[Leu], CAG[Gln], CGG[Arg], CGC[Arg], UCU[Ser], ACC[Thr], GUU[Val]), 10 of them ending in G/C. HEV-3 and HEV-4 shared very similar codon usage patterns. Both genotypes had identical preferred codons corresponding to 18 amino acids that had synonymous codons, of which eight preferred codons ended in G/C. In addition, HEV-1 had the largest number of preferred codons and overrepresented codons (Table 3), suggesting the highest codon usage bias. Consequently, both RSCU analysis and nucleotide composition suggested that the nucleotides at the third position in the codons limited the usage of preferred synonymous codons (Jenkins and Holmes, 2003).

**Table 3:** Number of preferred codons in the seven HEV genotypes.

	HEV-1	HEV-3	HEV-4	HEV-8	HEV-B	HEV-C1	HEV-C2
Number of preferred codons (RSCU > 1)	31	30	29	28	31	29	27
Number of preferred codons ending in G/C	20	16	15	14	16	18	19
Number of overrepresented codons (RSCU > 1.6)	11	4	1	6	1	1	1

**Figure 1:**
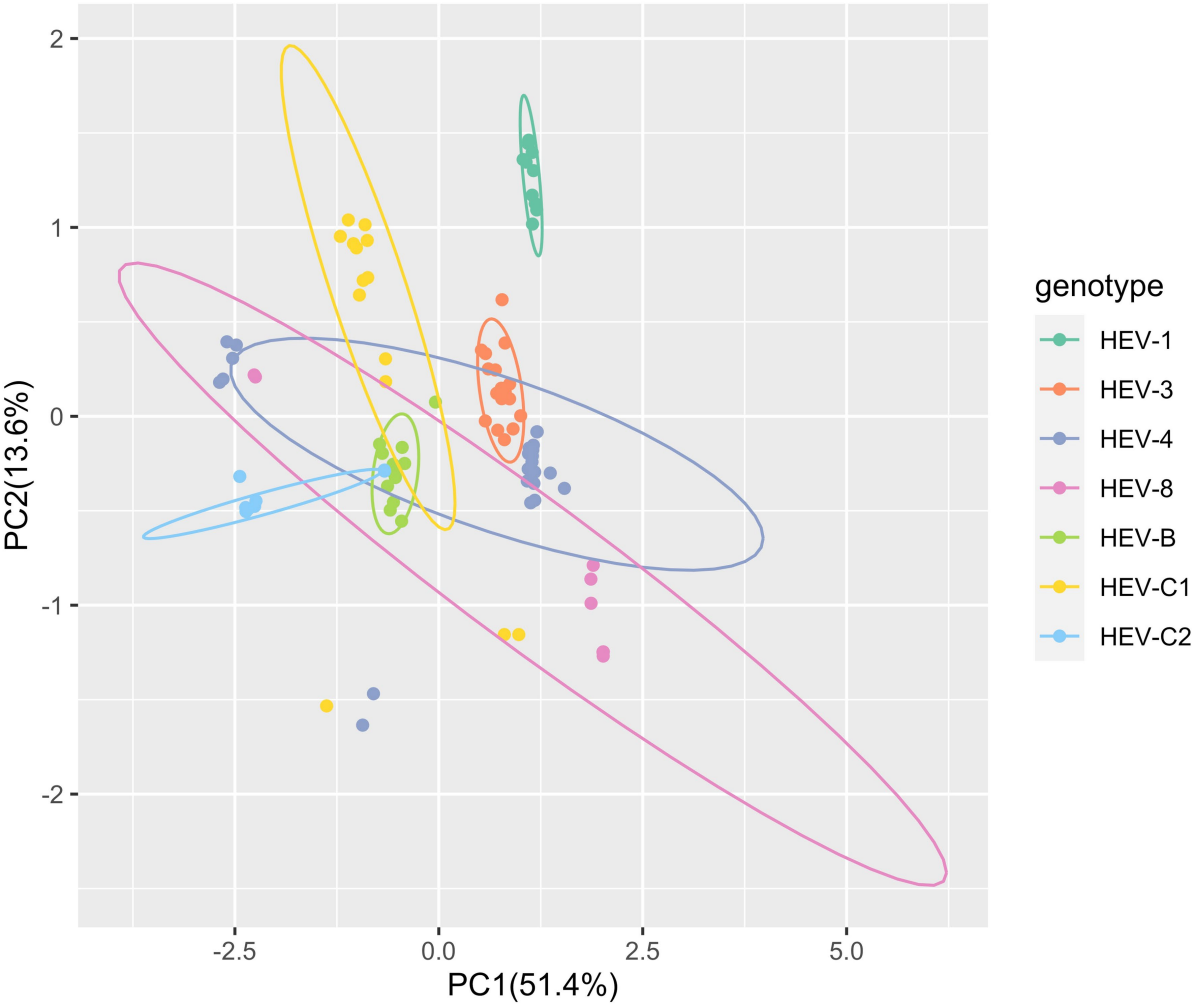
Principal component analysis (PCA) based on the hepatitis E virus (HEV) complete coding sequences. The first dimension was plotted against the second dimension. PCA plot showed the deviations and similarity among the 59 synonymous codons of 98 HEV sequences included in the study. Seven HEV genotypes were presented by colors. The ellipses in the figure predicted new observations with a probability of 0.95. New observations from the same group were expected to fall inside the ellipses.

Moreover, for the complete genomes, PCA based on the RSCU values of 59 codons showed that the first principal component explained 51.4% of the total variation and the second principal component explained 13.6% (Figure 1). Several clusters of the HEV complete genome sequences were observed, suggesting the genotype-specific codon usage patterns. However, predicting ellipses of HEV-4 and HEV-8 were large and overlapped with other genotypes. PCA for ORF1 and ORF2 provided similar findings (Supplementary Figure 1).

### Variation in the Dinucleotide Frequency That Affected the Codon Usage of HEV

Dinucleotide frequency may be affected by codon usage, mutation pressure, and natural selection (Khandia et al., 2019). We performed dinucleotide analysis of the seven HEV genotypes to understand the possible influence of dinucleotide frequency on codon usage. The dinucleotide frequency abundance of these HEV genotypes were not equal to theoretical frequency 1.0, and each dinucleotide had a different frequency, suggesting that HEV was affected by translation selection to form a unique dinucleotide usage pattern.

**Figure 2:**
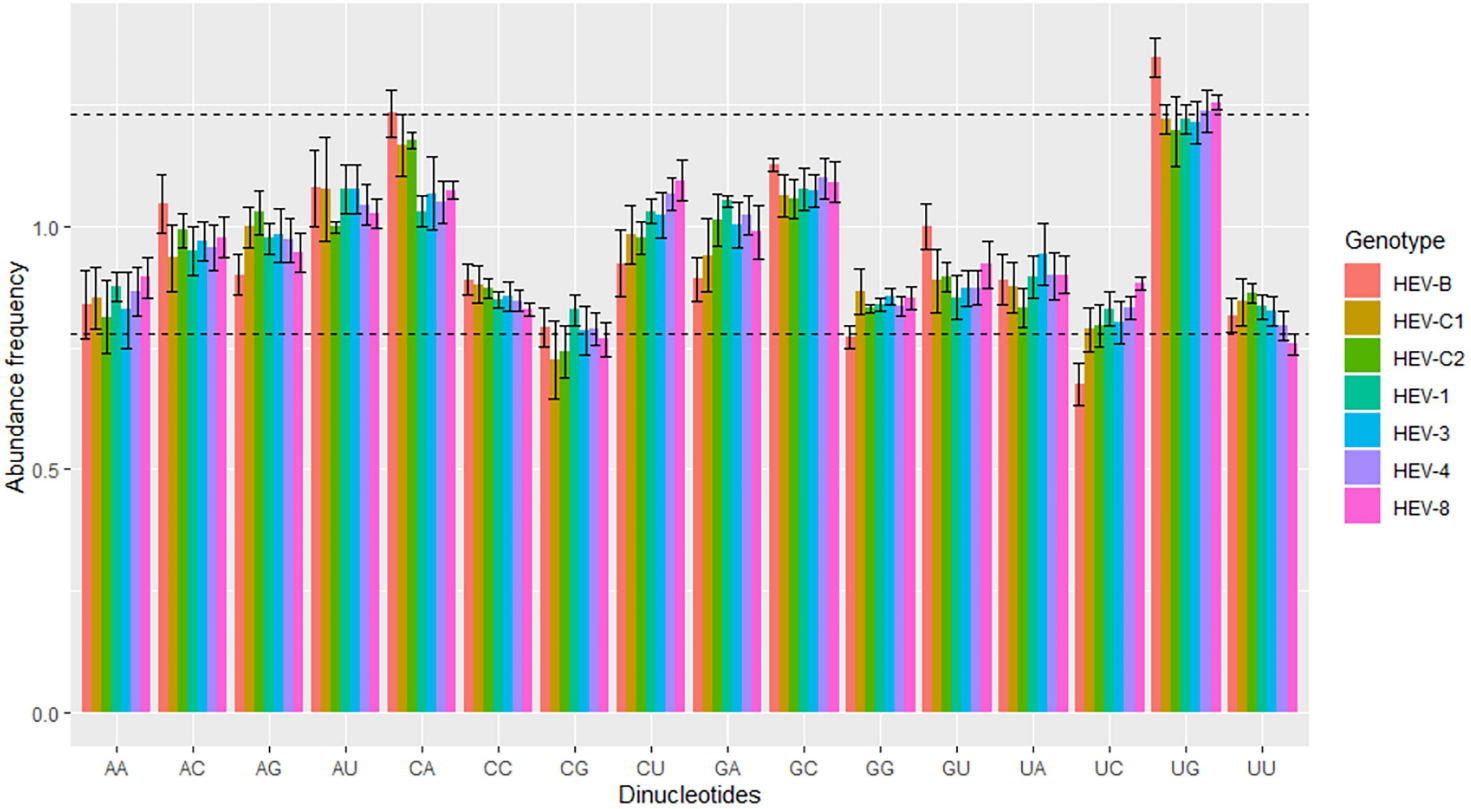
Dinucleotide abundance frequency based on the HEV complete coding sequences. The dashed lines showed overrepresented and underrepresented values. Seven HEV genotypes were presented by colors.

Generally, the dinucleotide frequencies of UpU, UpC, and CpG in HEV were low, while that of UpG was high. Furthermore, the dinucleotide usage pattern differed across the seven HEV genotypes. For the complete genomes, UpG was overrepresented in HEV-4, HEV-8 and HEV-B, CpG was underrepresented in HEV-C1 and HEV-C2, UpC was underrepresented in HEV-B, and UpU was underrepresented in HEV-8 (Figure 2). For ORF1, UpG was overrepresented in all seven genotypes, and CpG was underrepresented in the five genotypes except HEV-1 (Supplementary Figure 2). For ORF2, UpU was underrepresented in HEV-C1, HEV-4, and HEV-8; UpG was overrepresented in HEV-C1, HEV-C2, HEV-4, and HEV-8; CpA was overrepresented in HEV-C1; CpC was underrepresented in HEV-8; and CpG was underrepresented in HEV-C1 (Supplementary Figure 2). In contrast, for ORF3, HEV-B generally showed overrepresentation in dinucleotides; in other genotypes, ApA, ApU, CpG and GpC had higher dinucleotide frequencies, while ApG and UpA had lower frequencies compared with the complete genomes (Supplementary Figure 2).

### Identification of the Factors Affecting Codon Usage Pattern of HEV

**Figure 3:**
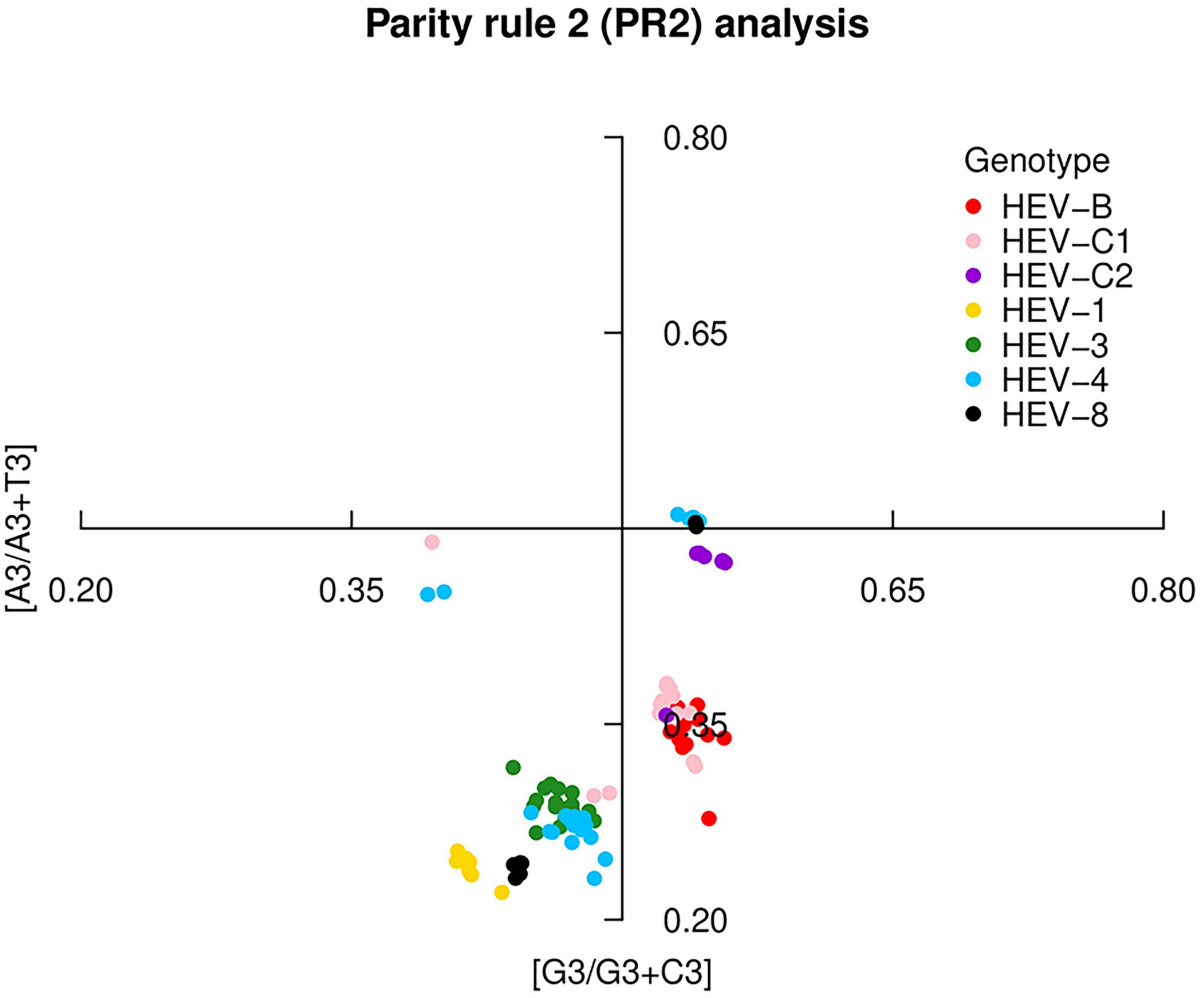
Parity Rule 2 (PR2) plot based on the HEV complete coding sequences. The center of the plot, where the value of both coordinates was 0.5, indicated no bias in mutation or selection rates. Seven HEV genotypes were presented by colors.

The PR2 analysis found significant deviations in codon usage of each genotype (A ≠ U and C ≠ G), indicating that mutation pressure and natural selection differed in the effect on the codon usage. HEV-C2 was closest to the center, indicating a low deviation between mutation pressure and natural selection (Figure 3). In addition, HEV-1, HEV-3, HEV-4, and HEV-8 preferably used the nucleotides U and C (pyrimidines) compared to A and G (purines). However, HEV-B, HEV-C1 and HEV-C2 preferred A to T. It showed an interspecies difference in the HEV codon usage pattern. PR2 analysis for ORF1, ORF2 and ORF3 was presented in Supplementary Figure 3.

Moreover, the ENC values of the complete genome, ORF1, ORF2 and ORF3 were evaluated to estimate the degree of codon usage bias in the coding sequences of the seven genotypes. In the complete genomes, the mean value of ENC of all genotypes was 53.5, which differed significantly across the HEV genotypes (*p* < 0.05). The largest ENC value was in HEV-C2 (57.3), followed by HEV-B (55.9). The smallest was in HEV-1 (48.9), followed by HEV-8 (51.4), HEV-3 (52.8), HEV-4 (53.3), and HEV-C1 (54.2). Both ORF1 and ORF2 showed the similar trend with the complete genomes; furthermore, the ENC value in ORF2 was slightly lower than that in ORF1. However, ORF3 showed different trend that HEV-C1 had lower ENC value than HEV-1 (*p* < 0.05).

**Figure 4:**
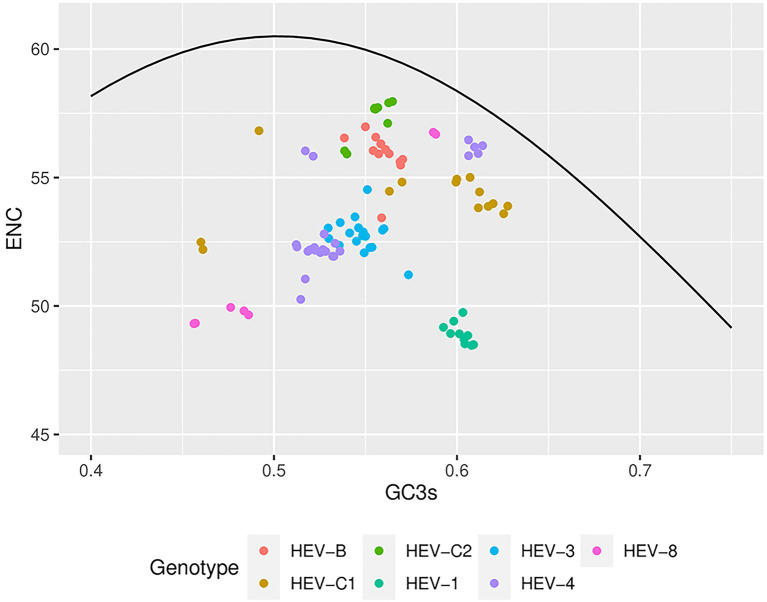
Effective number of codons (ENCs)-plot analysis based on the HEV complete coding sequences. ENC values were plotted against GC3s of the genotypes. The black line represented the standard curve when the codon usage bias was determined by only the GC3s composition. Seven HEV genotypes were presented by colors.

The theoretical curve in the ENC-plot represented the expected location of the gene when codon usage is determined solely by the mutation in GC3s. In the analysis for the complete genomes, HEV genotypes fell below the curve and clustered separately (Figure 4), suggesting that in addition to mutation pressure, natural selection also played an essential role in the codon usage bias. HEV-1, HEV-3, HEV-4, and HEV-8 were far away from the expected curve, suggesting that these genotypes were under greater natural selection. Moreover, significant correlation was found between ENC and various GC contents in HEV-4, HEV-8, HEV-C1 and HEV-C2 sequences, suggesting higher mutation pressure in these genotypes (Table 4; Chakraborty et al., 2019). The ENC-plot analysis for ORF1 and ORF2 were consistent with that of the complete genomes; however, error-free outliers were found in HEV-4 in ORF2, indicating that there was a large variation within the codon usage pattern of HEV-4. For ORF3, however, it showed that mutation pressure was dominant in HEV-B, HEV-C1, HEV-4, and HEV-8 (Supplementary Figure 4).

**Table 4:** Correlation analysis between ENC and GC contents of HEV complete coding sequences.

Genotype	ENC and GC%	ENC and GC1%	ENC and GC2%	ENC and GC3%	ENC and GC12%
HEV-1	−0.56	−0.5	0.37	−0.54	−0.16
HEV-3	−0.27	0.14	−0.33	−0.3	−0.09
HEV-4	−0.12	−0.91^**^	0.79^**^	0.77^**^	−0.27
HEV-8	0.03	−1^**^	−0.5	0.99^**^	−1^**^
HEV-B	−0.44	−0.39	0.3	−0.39	−0.26
HEV-C1	0.54^*^	0.79^**^	0.78^**^	0.21	0.81^**^
HEV-C2	−0.04	−0.98^**^	0.97^**^	0.88^**^	0.83^**^

** *p* < 0.01;

* *p* < 0.05.

**Figure 5:**
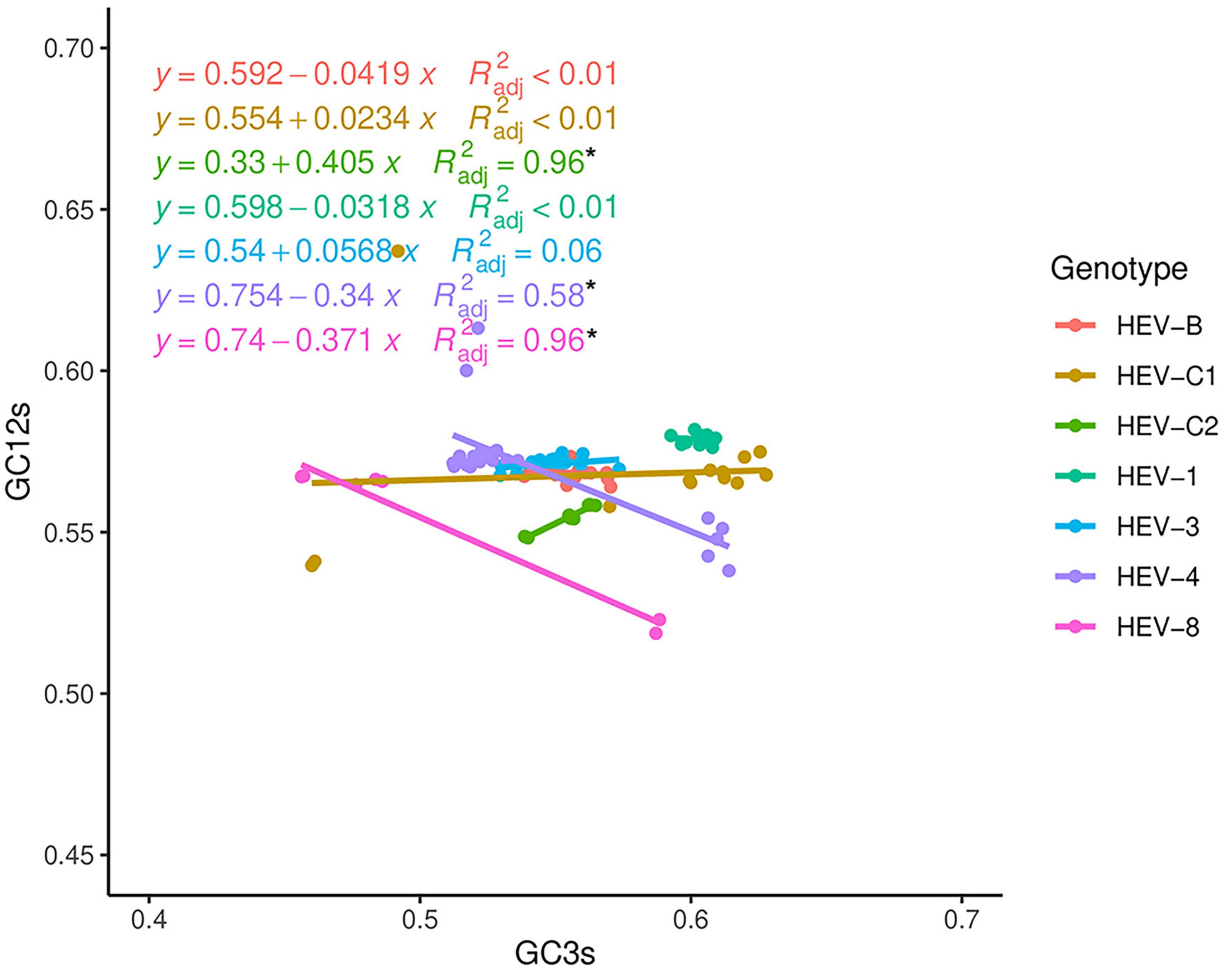
Neutrality analysis based on the HEV complete coding sequences. The correlation between GC content at first and second positions of codon (GC12s) and at third position of codon (GC3s) was calculated. The solid lines by colors represented the linear regression of GC12 against GC3s for the seven HEV genotypes. * Represented correlation significant at *p* < 0.05.

Neutrality analysis was used to further determine the effects of mutation pressure and natural selection on the codon usage bias of HEV. For the complete genomes, a significant correlation between GC3s and GC12s was observed in HEV-C2, HEV-4, and HEV-8 (*p* < 0.05; Figure 5), indicating mutation pressure played a role, though natural selection remained dominant with natural selection constraint ratios of 59.50%, 66.00%, and 62.90%, respectively. For the other genotypes, there was no significant correlation between GC3s and GC12s (*p* > 0.05), suggesting natural selection predominantly worked in their codon usage bias. Neutrality analysis for ORF1 and ORF2 were similar. However, for ORF3, it showed no significant correlation between GC3s and GC12s for HEV-8 (Supplementary Figure 5).

### Host Adaptation

By multiple comparisons, we found that RCDI values of ORF1 were significantly lower than those of ORF2 and ORF3 in HEV-1, HEV-3, HEV-4 and HEV-8. In contrast, in HEV-B, HEV-C1 and HEV-C2, RCDI values of ORF1 and ORF2 were similar and significantly lower than ORF3. It suggested that ORF1 had greater adaptation to hosts, compared to ORF2 and ORF3. In addition, the average RCDI values of ORF1, ORF2, and ORF3 in HEV-3 and HEV-4 with *Homo sapiens* and *Sus scrofa* showed no significant divergence, indicating similar adaptation to hosts (Table 5).

**Table 5:** Relative codon deoptimization index (RCDI) values of HEV ORF1, ORF2, and ORF3 coding sequences with different hosts.

HEV genotypes	Host	Coding sequences^a^			Average RCDI	*p*
		ORF1	ORF2	ORF3		
HEV-1	*Homo sapiens*	1.22	1.35	1.77	1.45	
HEV-3	*H. sapiens*	1.19	1.46	1.60	1.42	0.289
	*Sus scrofa*	1.23	1.42	1.49	1.38	
HEV-4	*H. sapiens*	1.20	1.44	1.73	1.46	0.932
	*S. scrofa*	1.26	1.54	1.59	1.46	
HEV-8	*Camelus bactrianus*	1.41	1.71	1.77	1.63	
HEV-B	*Gallus gallus*	1.18	1.14	1.69	1.34	
HEV-C1	*Rattus norvegicus*	1.20	1.21	2.12	1.51	
HEV-C2	*Mustela putorius furo*	1.30	1.33	1.66	1.43	

a The multiple comparisons using Tukey’s HSD test showed significant difference in the RCDI values among HEV coding sequences, except ORF2 and ORF3 in HEV-8, ORF1 and ORF2 in HEV-B, HEV-C1, and HEV-C2.

## Discussion

In this study, we collected the complete coding sequences of a total of 98 representative HEV genomes, covering seven human, zoonotic, and emerging animal genotypes, for a comprehensive analysis of codon usage bias. Our findings showed that the nucleotide composition of the seven HEV genotypes was similar; however, the proportion of GC3 in HEV-8 was lower than 50%, which slightly tended to use codon ending in A/U. In GC correlation analysis, the results showed that mutation pressure had played a vital role in shaping the HEV codon usage bias. In RSCU analysis, HEV genotypes clustered separately. HEV-3 and HEV-4 shared similar RSCU values and were clustered close to HEV-B and HEV-C1, suggesting that the codon usage bias of them may be influenced by different evolutionary drivers. Moreover, the effects of mutation pressure and natural selection were determined. Natural selection had a greater influence on codon usage bias of HEV genotypes. Among them, HEV-3 and HEV-4 were similarly affected. Some HEV-4 genomes were close to animal HEV (HEV-C1, HEV-C2, and HEV-B), while human HEV (HEV-1) was clustered separately, suggesting that natural selection differed widely across the HEV genotypes.

Generally, RNA viruses evolve by changing the composition of their genomes in response to changes in their host and environment (Streicker, 2013). As an important indicator of virus evolution, codon usage bias is influenced by various factors, including natural selection, mutation pressure, composition of the genome region, and gene length (Salim and Cavalcanti, 2008). Previous studies on porcine pestivirus and canine distemper virus have suggested that codon usage bias of those viruses was affected by natural selection using nucleotide composition comparison, PR2 analysis, ENC-plot and neutrality plot analysis (Pan et al., 2020; Wang et al., 2020). Therefore, we first characterized the nucleotide composition of HEV genotypes. Based on the complete genome, ORF1, ORF2, and ORF3, we found that HEV had an apparent preference for codons ending in G/C. Theoretically, when synonymous codon usage is affected only by mutation pressure, the frequencies of U and A nucleotides at the third codon position should be equal to those of G and C (van Hemert et al., 2016). Therefore, it concluded that natural selection had influence on the codon usage across all HEV genotypes.

Then, we estimated the RSCU values of 59 synonymous codons. Majority of the preferred codons (RSCU>1) in HEV genotypes were G/C ending. Furthermore, several distinct clusters were identified in PCA across the HEV genotypes, indicating different codon usage patterns. Therefore, HEV has a stable synonymous codon usage bias at the genotype level, though it is an RNA virus with a high mutation rate. Additionally, predicting ellipses of HEV-4 and HEV-8 were large and overlapped with other genotypes, which might be attributable to potential sampling and sequencing bias of sequences available in the GenBank. It warrants the improvement in sampling and sequencing of HEV to achieve a more global study.

Dinucleotide abundance affects codon usage bias in organisms (Khandia et al., 2019). For example, there were significant differences in dinucleotide abundance among genotypes of porcine astrovirus (Wu et al., 2020). In this study, a total of 16 dinucleotide abundance was obtained. Dinucleotide CpG was underrepresented, while dinucleotide UpG and CpA were overrepresented. Dinucleotide CpG is generally underrepresented in most viruses, including bluetongue virus and retrovirus (Karlin et al., 1994; Karlin and Burge, 1995). The exact mechanism of CpG underrepresentation in RNA viruses remained largely unknown, which are generally thought to be an attempt to evade host immune mechanisms and be affected by natural selection (Vetsigian and Goldenfeld, 2009; Khandia et al., 2019). The increase in CpA and UpG may be considered a compensation mechanism for the decrease in CpG and UpA (Yomo and Ohno, 1989; Rima and McFerran, 1997). Our findings showed that CpG and UpC were lower in several HEV genotypes, while UpG was higher, suggesting that dinucleotide abundance in HEV may be affected by translation selection.

Moreover, we performed PR2 analysis, ENC-plot, and neutrality analysis to better understand the role of mutation pressure and natural selection in shaping codon usage bias. Codon usage was moderately biased, which suggested that mutation pressure and natural selection played an unbalanced role in the formation of codon usage bias in the seven HEV genotypes. Previous studies have shown that ENC value is negatively correlated with gene expression (van Hemert and Berkhout, 2016). For example, vector-borne viruses such as the dengue virus have higher ENC values and lower codon usage, which may be more conducive to efficient replication in intermediate hosts (Jenkins and Holmes, 2003). Compared with the dengue virus, the ENC values of HEV were not low, and further differed among HEV genotypes. Low codon usage bias improves survival and efficient replication in the host environment, and reduces the energy required for viral biosynthesis and avoids competition with host protein synthesis (van Hemert et al., 2016). Furthermore, this study showed that natural selection and mutation pressure jointly influenced the codon usage bias in HEV-4, HEV-8, and HEV-C2, whereas natural selection predominantly worked in that of HEV-1, HEV-3, HEV-B, and HEV-C1.

Finally, RCDI values indicate the cumulative effects of codon usage bias on the expression of a gene, which is measured by comparing the codon usage of a virus with that of a host (Mueller et al., 2006). Our study found that HEV-3 and HEV-4 had similar RCDI values with *H. sapiens* and *S. scrofa*, suggesting that humans and swine had the same adaptability to zoonotic HEV. In addition, the RCDI value of ORF1 was generally low, indicating higher adaptation to hosts. Thus, from the perspective of codon adaptability, ORF2 and ORF3 may play a greater role in shaping cross-species transmission.

In this study, we concurrently included HEV complete genome sequences, ORF1, ORF2, and ORF3 for analysis, while did not include ORF4. So far, ORF4 has been identified in HEV-1 and HEV-C. However, it has not been found in other HEV genotypes or species. Currently, the findings on ORF4 function might be inconsistent. For HEV-1, it has been documented that ORF4 enhances the viral replication (Nair et al., 2016; Shafat et al., 2021). Furthermore, the expression of HEV-1 ORF4 increases viral replication of HEV-3 in cell culture (Yadav et al., 2021). In contrast, for HEV-C, ORF4 is unnecessary for viral replication (Tanggis et al., 2018). Therefore, it warrants further study for clarification of ORF4 function. In addition, the findings based on HEV complete genomes were similar to those of ORF1 and ORF2, while that of ORF3 was inconsistent. This disparity may be attributable to the short length of ORF3 (approximately 100 amino acids); furthermore, some of the 59 synonymous codons did not present in each HEV genome, resulting in larger or smaller codon usage bias, such as Phe not being used in MG976720. Subsequently, a few codon usage differences would result in large variation in the analysis. Compared with previous studies (Baha et al., 2019), we excluded HEV-2, 5, 6, and 7 that had 1–3 complete genomes available in the GenBank, and additionally included HEV-B and HEV-C to study the differences among HEV species. Moreover, in addition to the three ORFs, we performed a comprehensive analysis of HEV complete coding sequences, which could obtain a conclusion on the whole genome level.

## Strength and Limitation

This study is of great significance. First, viruses obtain adaption to replicate by controlling the expression of proteins. By optimizing their genomic codon usage bias, some viruses achieve a high replication rate or evade the host immune system (Costafreda et al., 2014; Bellare et al., 2015). Based on the control of viral protein expression, codon usage bias may be utilized to provide new ideas for HEV vaccine design. Second, codon usage bias provides theoretical evidence for further research on transcriptional regulation, function, and pathological correlation of HEV protein, which may facilitate better understanding the infection and pathogenesis of HEV. Third, information on the codon usage patterns of HEV genotypes may help identify potential animal hosts and laboratory animal models for studying pathogenesis and vaccines.

There are also some limitations in this study. First, some HEV genotypes had limited sequences available in the GenBank database, which might not accurately characterize the codon usage bias of the genotypes. However, we included all available reference sequences for each HEV genotype, so that our findings remained significant. Second, although the overall RSCU value can reveal the codon usage bias in the genome, it may hide the difference in codon usage among the genes in the genome (Hassan et al., 2009). Third, we did not perform experimental study to validate the findings in the bioinformatics analysis. However, characterization of codon usage pattern may provide preliminary evidence and facilitate the identification of main drivers affecting codon usage bias. It has been documented that it is reasonable to infer the evolutionary driving force that shapes codon usage pattern, such as Nipah virus (Chakraborty et al., 2019), H1N1/pdm2009 (Guo et al., 2020), and bluetongue virus (Yao et al., 2020), from a codon usage perspective and using simply bioinformatics methodology.

## Conclusion

HEV genomes had different codon usage bias, though they shared similar nucleotide composition. In terms of RSCU values, there were obvious difference in codon usage bias among human, zoonotic, and animal HEV genotypes, and also variations within certain genotypes such as HEV-4, HEV-8, and HEV-C1. Moreover, natural selection and mutation pressure were the main drivers affecting the formation of HEV codon usage bias. Both of them jointly influenced the codon usage bias in HEV-4, HEV-8, and HEV-C2, whereas natural selection predominantly worked in that of HEV-1, HEV-3, HEV-B and HEV-C1. This study may provide new insights into the evolution of HEV in terms of codon usage bias.

## Data Availability Statement

The datasets presented in this study can be found in online repositories. The names of the repository/repositories and accession number(s) can be found in the article/Supplementary Material.

## Author Contributions

YL: conceptualization. YL and ZM: methodology and writing—review and editing. LZ and LH: software. BL and HW: formal analysis and writing—original draft preparation. BL, LZ, LH, and HW: data curation. All authors contributed to the article and approved the submitted version.

## Funding

This work was supported by the National Natural Science Funds of China (grant number 81973101) and the Science Technology Department of Zhejiang Province (grant number LGF20H190002). The funding organization had no role in the design and conduct of the study; collection, management, analysis, and interpretation of the data; preparation, review, or approval of the manuscript; or decision to submit the manuscript for publication.

## Conflict of Interest

The authors declare that the research was conducted in the absence of any commercial or financial relationships that could be construed as a potential conflict of interest.

## Publisher’s Note

All claims expressed in this article are solely those of the authors and do not necessarily represent those of their affiliated organizations, or those of the publisher, the editors and the reviewers. Any product that may be evaluated in this article, or claim that may be made by its manufacturer, is not guaranteed or endorsed by the publisher.

## Footnotes

^1^ https://www.ncbi.nlm.nih.gov

^2^ https://www.bioinformatics.nl/emboss-explorer/

^3^ https://www.R-project.org/

^4^ https://www.bioinformatics.nl/emboss-explorer/
