# Supplementary material for: Codon Usage of Hepatitis E Viruses: A Comprehensive Analysis
Source: Front Microbiol. 2022 Jun 21;13:938651. doi: 10.3389/fmicb.2022.938651 (PMC9253588; doi:10.3389/fmicb.2022.938651)
Supplement: Supplementary file 1 [file Table_1.docx]

Supplementary Table 1. Accession numbers of the HEV genomes included in the study

| **Genotype** | **Accession numbers** |
| --- | --- |
| HEV-B | AY535004 |
| HEV-B | EF206691 |
| HEV-B | GU954430 |
| HEV-B | JN597006 |
| HEV-B | KC454286 |
| HEV-B | KF511797 |
| HEV-B | KM377618 |
| HEV-B | MG976720 |
| HEV-B | MN562265 |
| HEV-B | MW589651 |
| HEV-B | MW924815 |
| HEV-B | OM037109 |
| HEV-B | MZ736614 |
| HEV-B | MW589652 |
| HEV-C1 | MN450851 |
| HEV-C1 | MN450852 |
| HEV-C1 | MN450853 |
| HEV-C1 | MN450854 |
| HEV-C1 | MN450855 |
| HEV-C1 | MW149254 |
| HEV-C1 | KM516906 |
| HEV-C1 | NC_038504 |
| HEV-C1 | GU345042 |
| HEV-C1 | MK050105 |
| HEV-C1 | MG020023 |
| HEV-C1 | MG020024 |
| HEV-C1 | MG020025 |
| HEV-C1 | MG020022 |
| HEV-C2 | LC177788 |
| HEV-C2 | LC177789 |
| HEV-C2 | LC177790 |
| HEV-C2 | LC177791 |
| HEV-C2 | LC177792 |
| HEV-C2 | AB890001 |
| HEV-C2 | AB890374 |
| HEV-C2 | LC057247 |
| HEV-C2 | JN998606 |
| HEV-C2 | JN998607 |
| HEV-1 | M73218 |
| HEV-1 | L08816 |
| HEV-1 | X98292 |
| HEV-1 | AY230202 |
| HEV-1 | AY204877 |
| HEV-1 | JF443721 |
| HEV-1 | LC225387 |
| HEV-1 | FJ457024 |
| HEV-1 | MH918640 |
| HEV-1 | JF443726 |
| HEV-3 | AF082843 |
| HEV-3 | AP003430 |
| HEV-3 | FJ705359 |
| HEV-3 | AB248521 |
| HEV-3 | AB369687 |
| HEV-3 | AF455784 |
| HEV-3 | JQ013794 |
| HEV-3 | FJ998008 |
| HEV-3 | AY115488 |
| HEV-3 | AB369689 |
| HEV-3 | JQ953664 |
| HEV-3 | KU513561 |
| HEV-3 | AB290313 |
| HEV-3 | MF959765 |
| HEV-3 | LC260517 |
| HEV-3 | MK390971 |
| HEV-3 | MF959764 |
| HEV-3 | KP294371 |
| HEV-3 | FJ906895 |
| HEV-4 | AB074915 |
| HEV-4 | AB108537 |
| HEV-4 | AB197673 |
| HEV-4 | AB220974 |
| HEV-4 | AB369688 |
| HEV-4 | AB369690 |
| HEV-4 | AB909124 |
| HEV-4 | AB909125 |
| HEV-4 | AJ272108 |
| HEV-4 | AY723745 |
| HEV-4 | DQ279091 |
| HEV-4 | EU676172 |
| HEV-4 | FJ610232 |
| HEV-4 | GU119961 |
| HEV-4 | HM152568 |
| HEV-4 | JQ655733 |
| HEV-4 | KC692453 |
| HEV-4 | KC163335 |
| HEV-4 | KJ155502 |
| HEV-4 | KR872414 |
| HEV-4 | KY929404 |
| HEV-4 | MF567575 |
| HEV-4 | MK410048 |
| HEV-8 | MH410174 |
| HEV-8 | MH410175 |
| HEV-8 | MH410176 |
| HEV-8 | KX387865 |
| HEV-8 | KX387866 |
| HEV-8 | KX387867 |
| HEV-8 | LC597024 |
| HEV-8 | LC597025 |

Supplementary Table 2. Nucleotide compositions of ORF1, ORF2 and ORF3 in the seven HEV genotypes.

| **Genotype** | **GC1*** | **GC2** | **GC3** | **A3s** | **U3s** | **C3s** | **G3s** |
| --- | --- | --- | --- | --- | --- | --- | --- |
| **ORF1** |  |  |  |  |  |  |  |
| HEV-B | 65.95±0.46 | 47.07±0.2 | 55.66±0.97 | 17.15±1.66 | 34.95±1.09 | 30.33±0.73 | 38.29±0.89 |
| HEV-C1 | 63.42±1.35 | 47.98±1.32 | 57.96±5.87 | 17.09±2.01 | 32.68±6.01 | 33.13±2.60 | 38.23±4.87 |
| HEV-C2 | 54.76±2.58 | 53.89±3.15 | 55.05±1.29 | 22.52±1.35 | 28.67±3.75 | 28.34±1.04 | 37.19±1.02 |
| HEV-1 | 64.45±0.26 | 49.45±0.21 | 60.70±0.79 | 10.62±0.31 | 34.64±0.86 | 42.44±0.88 | 30.60±0.53 |
| HEV-3 | 63.90±0.55 | 48.72±0.34 | 55.19±1.53 | 15.75±0.96 | 36.55±1.45 | 35.37±1.55 | 31.89±1.08 |
| HEV-4 | 61.72±4.67 | 51.23±4.47 | 54.42±3.60 | 18.11±3.56 | 34.54±7.39 | 32.92±1.37 | 32.21±3.81 |
| HEV-8 | 60.41±4.95 | 48.98±1.12 | 50.41±5.48 | 16.78±3.32 | 40.00±10.54 | 30.99±1.08 | 29.16±5.07 |
| **ORF2** |  |  |  |  |  |  |  |
| HEV-B | 61.67±0.77 | 52.24±0.2 | 54.68±1.7 | 18.41±0.79 | 33.54±1.81 | 33.37±2.17 | 31.16±2.07 |
| HEV-C1 | 61.47±1.34 | 51.26±0.24 | 57.13±5.79 | 16.02±1.1 | 33.02±6.22 | 34.19±3.21 | 33.63±3.58 |
| HEV-C2 | 56.23±2.38 | 54.65±2.02 | 55.60±1.51 | 24.65±4.47 | 25.71±6.15 | 30.00±1.08 | 35.85±2.69 |
| HEV-1 | 63.70±0.30 | 53.75±0.14 | 56.89±0.89 | 10.81±0.69 | 37.54±1.15 | 40.13±0.97 | 25.72±0.64 |
| HEV-3 | 61.74±0.59 | 53.45±0.30 | 50.69±1.26 | 13.43±1.20 | 42.01±0.86 | 33.44±1.06 | 25.74±1.15 |
| HEV-4 | 60.30±2.49 | 52.72±3.03 | 50.85±5.47 | 15.85±5.7 | 39.54±11.45 | 32.40±3.12 | 27.02±5.70 |
| HEV-8 | 60.08±2.29 | 51.75±3.44 | 45.19±7.35 | 16.87±5.76 | 44.45±14.18 | 29.65±1.17 | 22.66±7.34 |
| **ORF3** |  |  |  |  |  |  |  |
| HEV-C1 | 64.53±3.1 | 68.06±4.16 | 71.52±1.89 | 14.24±3.57 | 18.79±3.37 | 41.58±2.4 | 42.93±1.06 |
| HEV-C2 | 68.37±3.57 | 66.2±2.28 | 61.94±2 | 15.88±4.52 | 27.85±6.09 | 41.44±0.35 | 30.39±3.15 |
| HEV-1 | 64.49±0.23 | 51.27±0.16 | 60.23±0.52 | 10.87±0.39 | 34.46±0.67 | 41.77±0.69 | 29.7±0.46 |
| HEV-3 | 63.45±0.42 | 50.77±0.27 | 54.81±1.09 | 14.98±0.73 | 37.03±1.08 | 35.2±1.13 | 30.55±0.79 |
| HEV-4 | 70.3±3.57 | 64.63±5.35 | 72.23±4.4 | 10.76±1.36 | 19.48±4.36 | 44.37±1.8 | 36.37±5.64 |
| HEV-8 | 68.39±0.67 | 62.28±0.66 | 69.86±1.4 | 12.37±0.87 | 20.49±1.55 | 42.2±1.06 | 36.06±1.63 |

*The values in the cells were represented as mean% ± SD%.


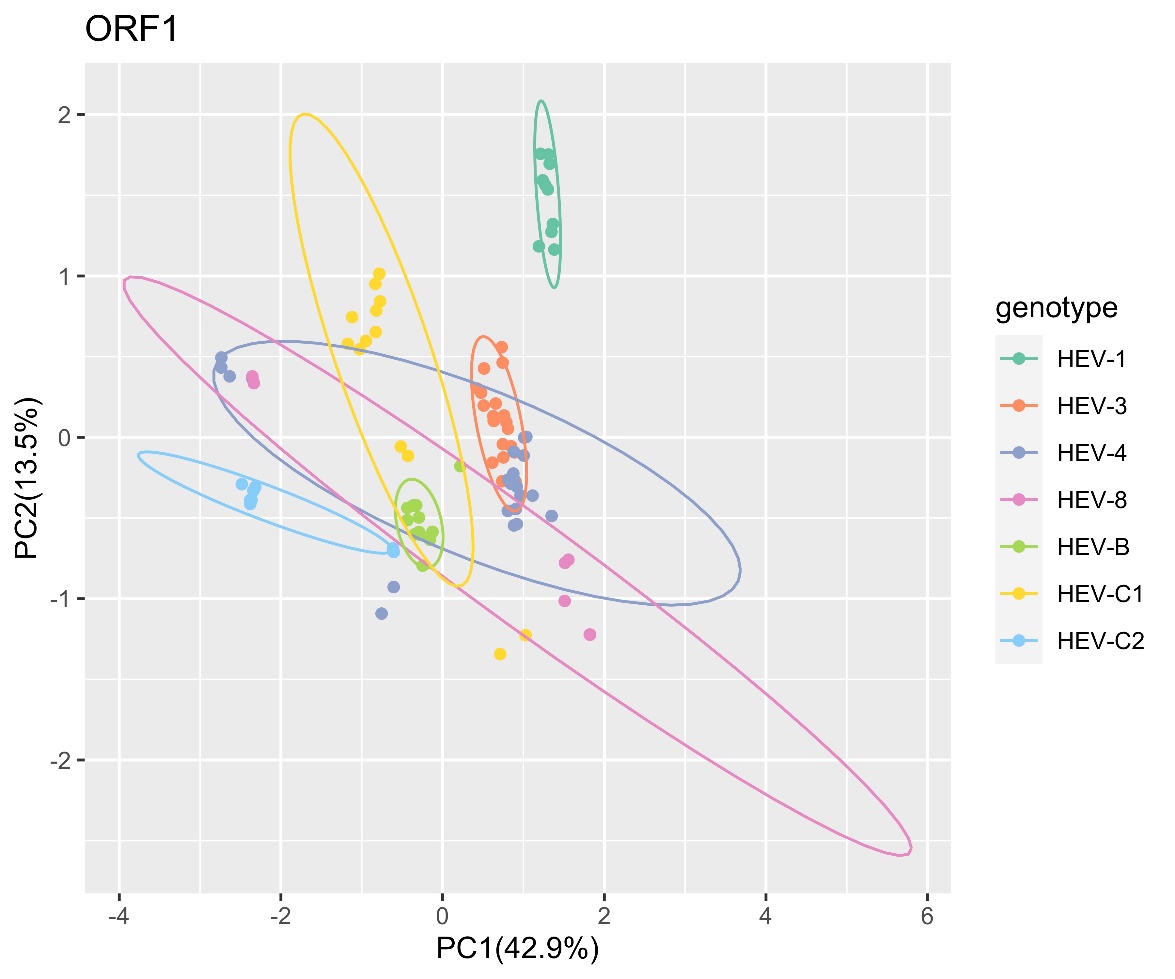


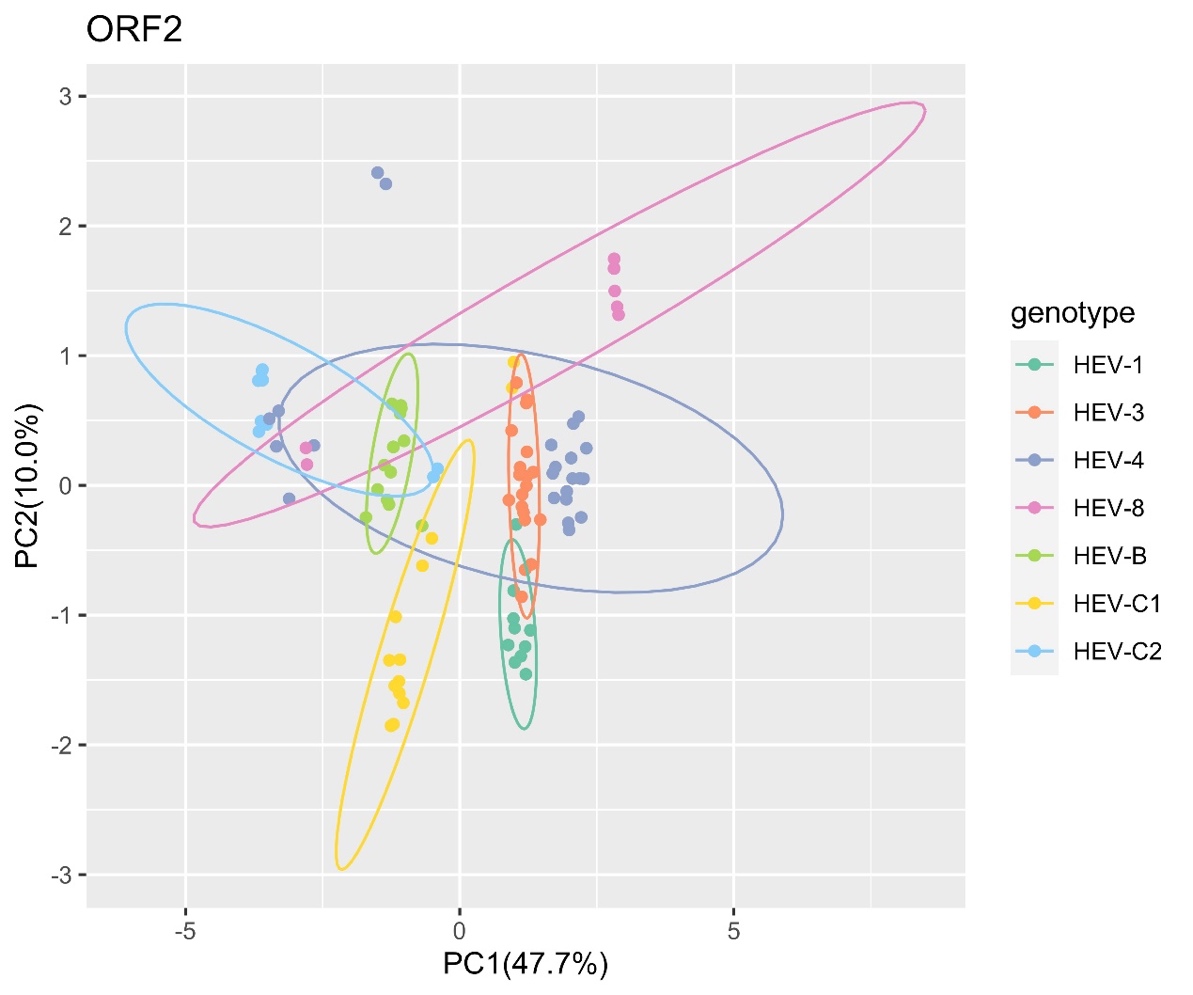


Supplementary Fig 1. Principal component analysis (PCA) based on the HEV ORF1, and ORF2 coding sequences. The first dimension was plotted against the second dimension. PCA plot showed the deviations and similarity among the 59 synonymous codons of 98 HEV sequences included in the study. Seven HEV genotypes were presented by colors. The ellipses in the figure predicted new observations with a probability of 0.95. New observations from the same group were expected to fall inside the ellipses.


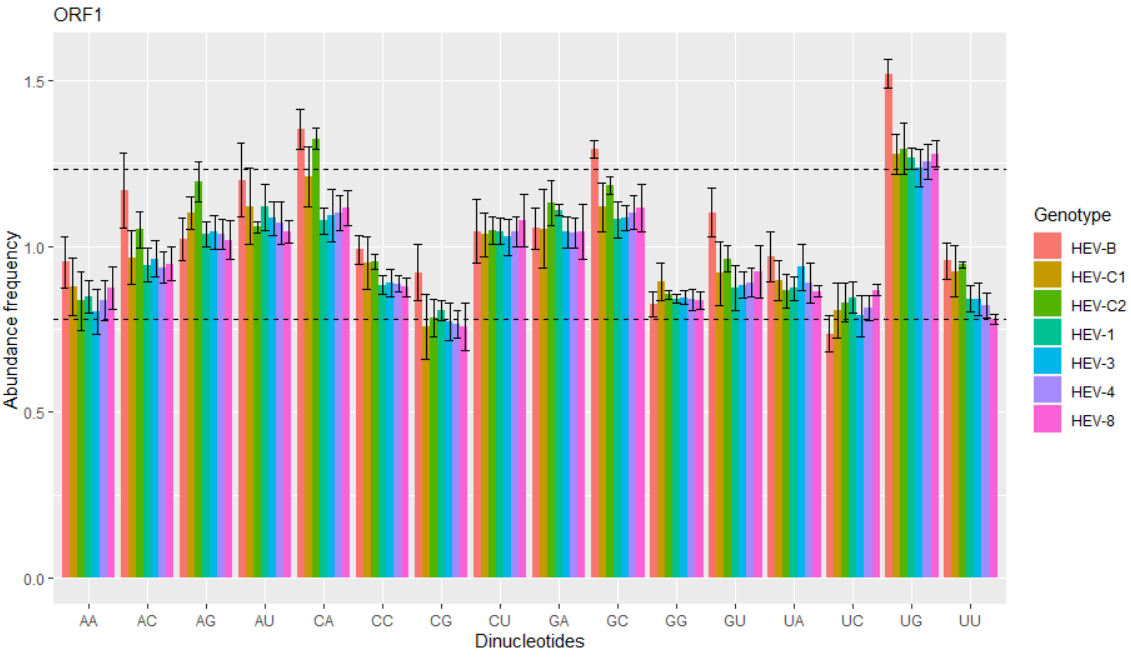


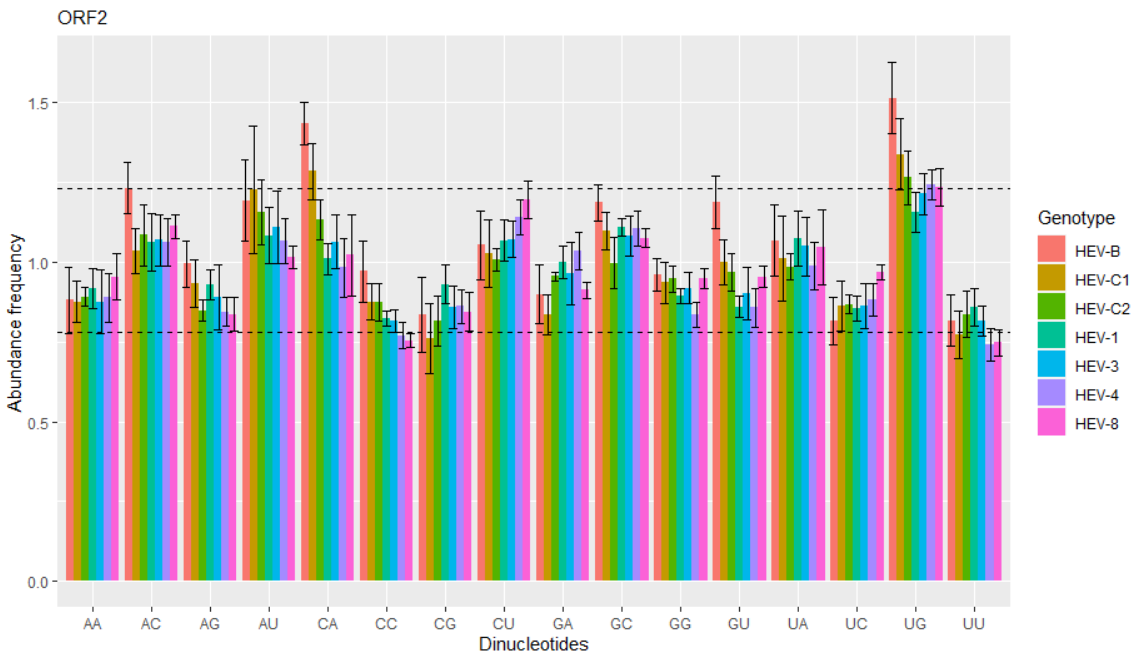


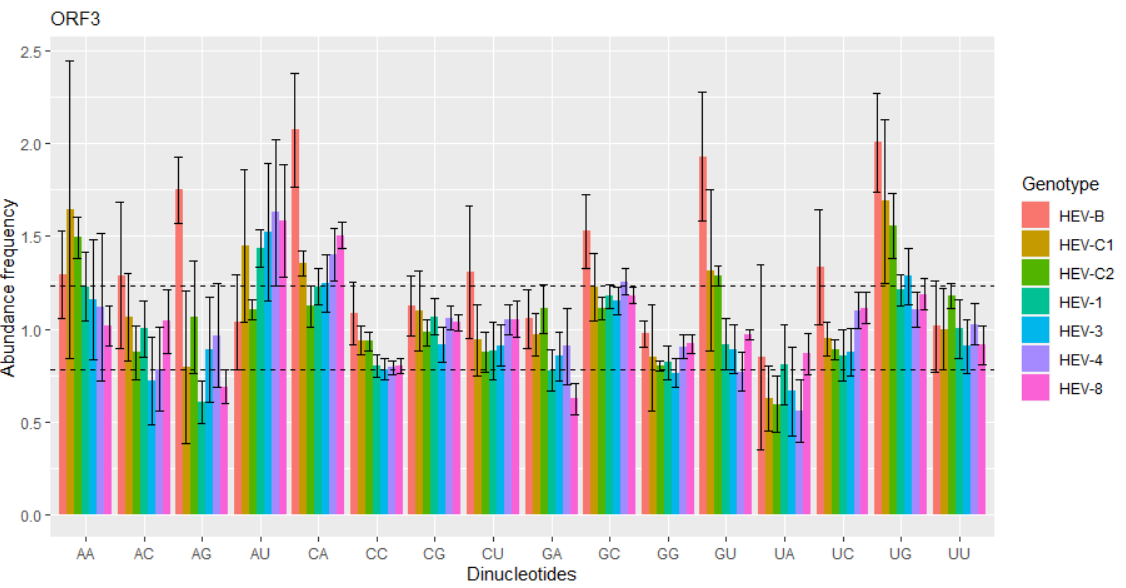


Supplementary Fig 2. Dinucleotide abundance frequency based on the HEV ORF1, ORF2 and ORF3 coding sequences. The dashed lines showed overrepresented and underrepresented values. Seven HEV genotypes were presented by colors.


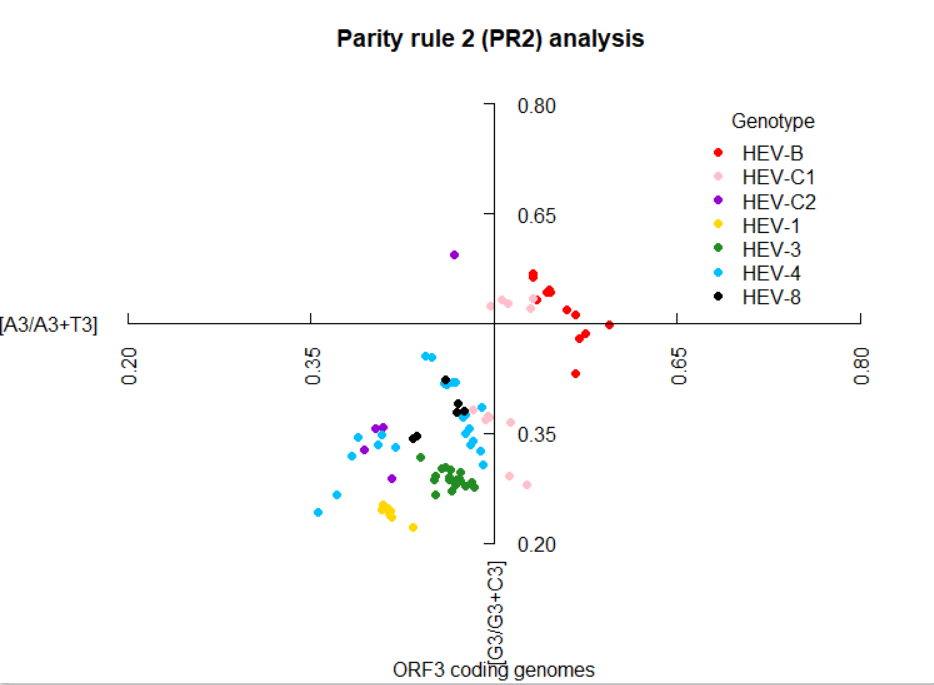

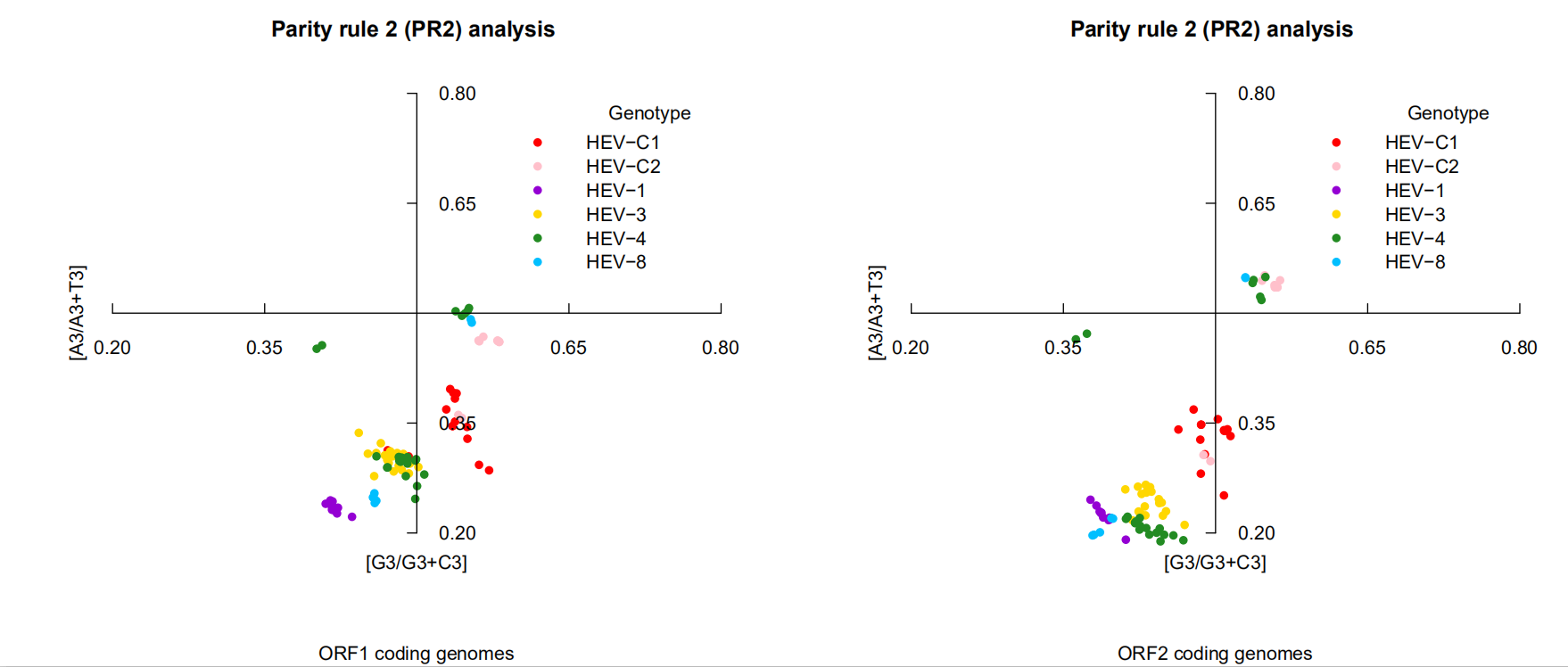


Supplementary Fig 3. Parity Rule 2 (PR2) plot based on the HEV ORF1, ORF2, and ORF3 coding sequences. The center of the plot, where the value of both coordinates was 0.5, indicated no bias in mutation or selection rates. Seven HEV genotypes were presented by colors.


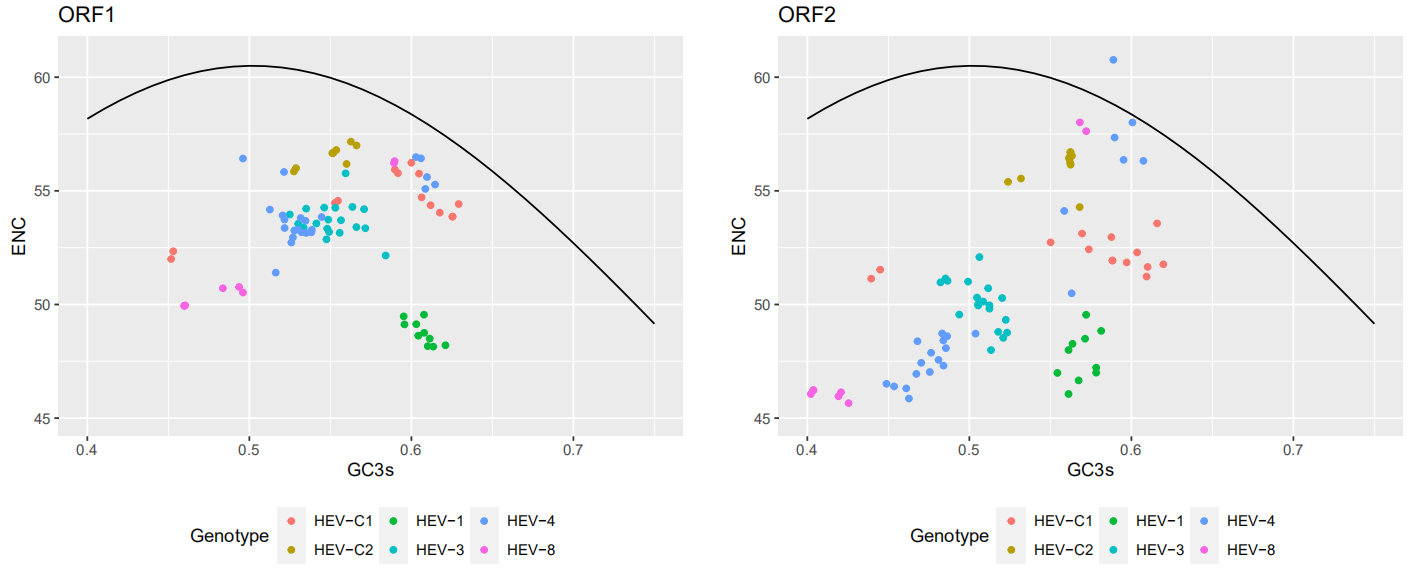


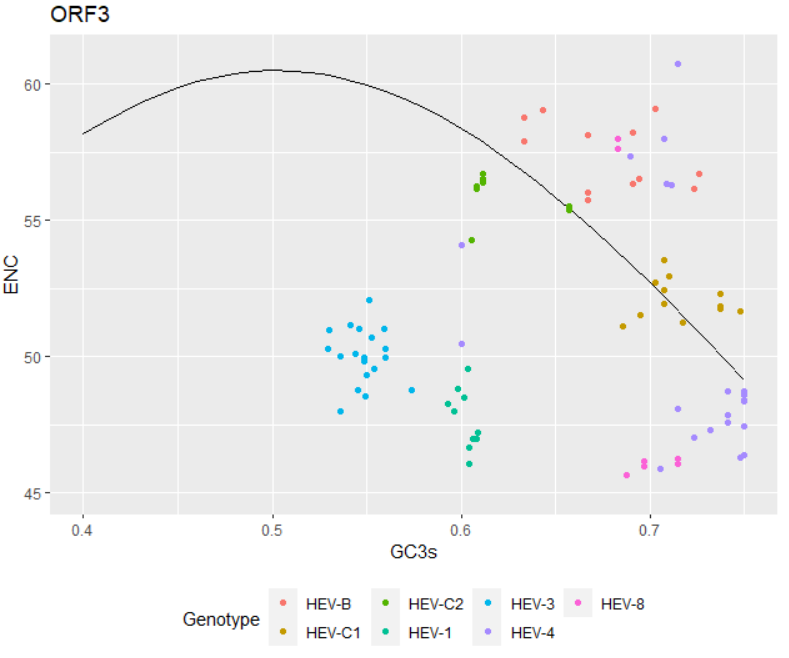


Supplementary Fig 4. Effective codon number (ENC)-plot analysis of the HEV ORF1, ORF2, and ORF3 coding sequences, with ENC values plotted against GC3s of the genotypes. The black line represented the standard curve when the codon usage bias was determined by only the GC3s composition. Seven HEV genotypes were presented by colors.


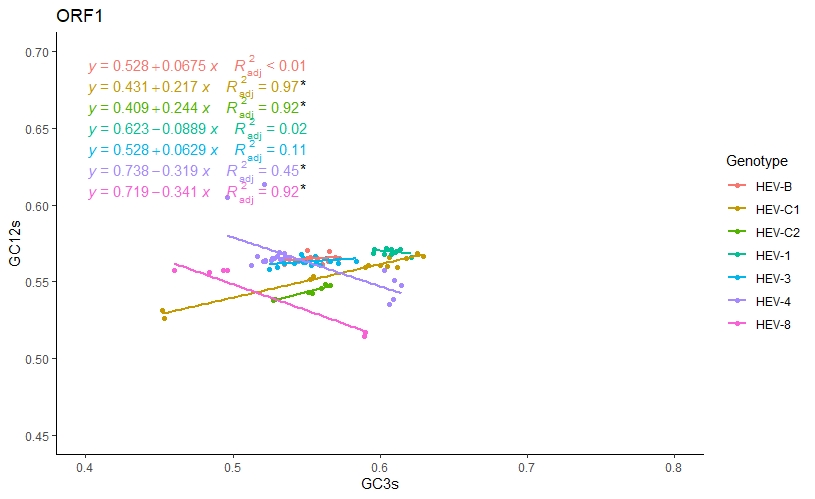


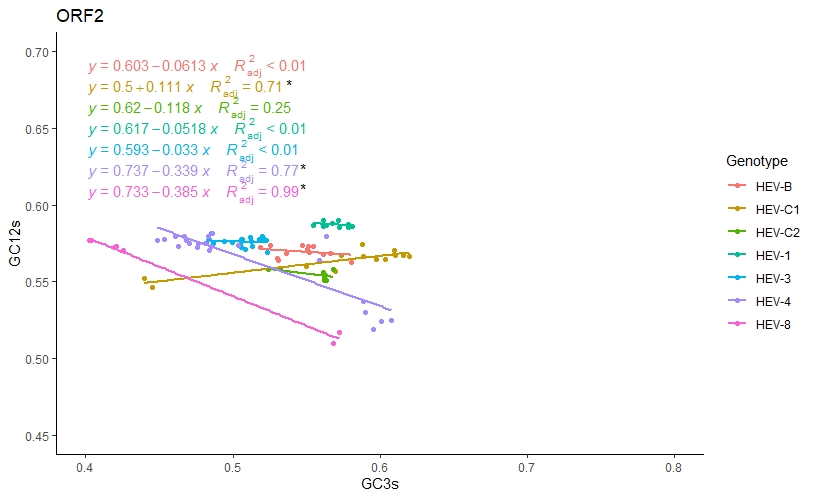


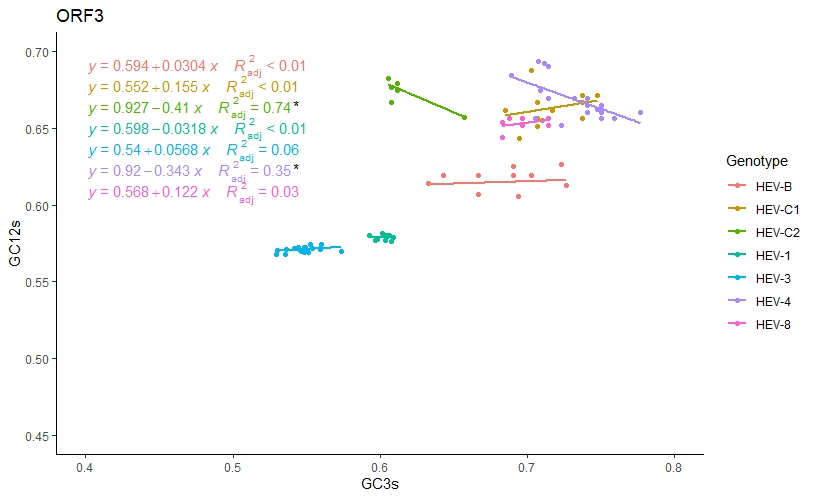


Supplementary Fig 5. Neutrality analysis based on the HEV ORF1, ORF2, and ORF3 coding sequences displayed the correlation between GC content at first and second positions of codon (GC12s) and at third position of codon (GC3s). The solid lines by colors represented the linear regression of GC12s against GC3s for the seven genotypes. * Represented correlation significant at P<0.05.
